# DEPRESSION IS ASSOCIATED WITH POORER CLINICAL FUNCTIONING AMONG HISPANIC OLDER ADULTS

**DOI:** 10.1093/geroni/igac059.1865

**Published:** 2022-12-20

**Authors:** Andres Duarte, Dilianna Padron, Joanna Gonzalez, Patricia Garcia, Lisandra Mendoza, Ranjan Duara, Miriam Rodriguez

**Affiliations:** Albizu University, Miami, Florida, United States; Albizu University, Miami, Florida, United States; Mt. Sinai Medical Center, Miami Beach, Florida, United States; Rehabilitation Hospital of Indiana, Indianapolis, Indiana, United States; Department of Veterans Affairs Bay Pines, Cape Coral, Florida, United States; Mt. Sinai Medical Center, Miami Beach, Florida, United States; Albizu University, Miami, Florida, United States

## Abstract

**Objective:**

The aim of the current study was to examine associations between depression and clinical functioning among a multi-ethnic sample.

**Methods:**

35 cognitively normal and Mild Cognitive Impairment (MCI) participants were included and self-identified as Hispanic or white non-Hispanic (WNH). The Hispanic group (n=18), had a mean age of 70.83 (SD=7.66) and 15.59 mean years of education (SD= 3.43). The WNH group (n=17) had a mean age of 71.76 (SD=6.9) and 16.81 mean years of education (SD=2.59). Subjects were given the Alzheimer’s disease Cooperative Study (ADCS) ADL inventory, the modified Clinical Dementia Rating scale (mCDR), and the Geriatric Depression Scale (GDS). Linear regressions were conducted to analyze the predictive associations between GDS scores and ADL functioning while controlling for the effect of diagnosis and age.

**Results:**

Among Hispanics, the overall regression was significant (R2 = .622, F(2,17)= 12.32, p<.001). Higher GDS scores was found to significantly predict worse mCDR scores (β = .676, p<.001) when controlling for the effects other factors. When examining ADCS-ADL scores, the overall model was also found to be significant (R2= .413, F(2,17)=5.28, p<.05). Higher GDS scores significantly predicted worse ADCS-ADL scores (β = =.652, p<.01) when controlling for the effects of other factors. Diagnosis and age did not significant predict ADL scores. Among the WNH group, the regression model was not significant and depression was not a significant predictor of ADL functioning.

**Conclusions:**

The results suggest Hispanics are more vulnerable to the effects of depression on ADL function which has important implications for AD diagnosis.

